# Yinhua Gouteng decoction alleviates tic disorder symptoms by modulating neuro-inflammation in a rat model

**DOI:** 10.3389/fimmu.2025.1680975

**Published:** 2026-02-04

**Authors:** Mingge Hu, Yan Yang, Shuxia Wang, LiRong Huang, Lifei Chen, Xinguang Zhang, Wenbo Yao, Zheng Xue

**Affiliations:** 1 Shanghai Municipal Hospital of Traditional Chinese Medicine, Shanghai University of Traditional Chinese Medicine, Shanghai, China; 2 Key Laboratory of Organ Regeneration and Transplantation of Ministry of Education, Institute of Immunology, The First Hospital, Jilin University, Changchun, China

**Keywords:** dopamine, gut microbiota, neuroinflammation, tic disorders, Yinhua Gouteng decoction

## Abstract

**Background:**

Tic disorders (TDs) are childhood-onset neurodevelopmental disorders characterized by complex neurochemical dysregulation, and inflammation plays a critical role in TD pathogenesis. Yinhua Gouteng Decoction (YHGTD), a traditional Chinese medicinal formula, has demonstrated clinical efficacy in TD management. However, the specific pharmacological mechanisms underlying its effects remain unclear. In this study, we investigated the neuroprotective and anti-inflammatory effects of YHGTD in a 3,3′-iminodipropionitrile (IDPN)-induced TD rat model.

**Methods:**

A rat model of IDPN-induced was established. Behavioral assessments, striatal histopathology, and quantification of striatal dopamine (DA) levels and dopamine receptor (DR) expression were measured to assess the effects of YHGTD on tic symptoms and dopaminergic function. Microglial activation was examined by immunofluorescence staining, while IL-1*β*, TNF-*α*, and IL-6 in serum, striatum, and colon were quantified using ELISA or qPCR. In addition, 16S rRNA sequencing was used to analyze alterations in the gut microbiota composition. Western blotting was performed to assess TLR4/MyD88/NF-*κ*B pathway activation in the striatum and colon.

**Results:**

YHGTD significantly improved motor and stereotypical behaviors in TD rats, decreased spontaneous activity, total travel distance, prolonged rest time, and normalized movement trajectories. It attenuated striatal neuropathology, elevated DA levels, and downregulated the expression of DRD1 and DRD2. YHGTD also suppressed microglial activation and reduced the levels of IL-1β, TNF-α, and IL-6 in the striatum, serum, and colon. Furthermore, YHGTD restored gut microbial homeostasis and reduced the abundance of proinflammatory bacterial taxa. Finally, we found that YHGTD downregulated the TLR4/MyD88/NF-κB signaling pathway in both the striatum and colon.

**Conclusion:**

YHGTD alleviated TD symptoms through neuroprotective and anti-inflammatory mechanisms, accompanied by alterations in the microbiota composition, supporting its potential as a therapeutic option for TD.

## 1 Introduction

Tic disorders (TDs) are chronic neurodevelopmental disorders characterized by involuntary movements (motor tics) and vocalizations (phonic tics) (1). Approximately 6% of children worldwide are affected by TD, showing a notable sex disparity, with a male-to-female ratio of approximately 4:1 (2, 3). Around 85.7% of individuals with TD exhibit psychiatric comorbidities, with 57.7% presenting two or more coexisting conditions, most frequently attention-deficit/hyperactivity disorder and obsessive-compulsive disorder (4). In approximately 23% of cases, symptoms persist into adulthood, significantly impairing psychological well-being and social adaptation (5). Dysfunction of the dopaminergic system is a key contributor to TD pathogenesis (6, 7), and dopamine D2 receptor (DRD2) antagonists, such as tiapride and haloperidol, are among the most effective pharmacological agents (8–10). However, its clinical utility is constrained by significant adverse effects, such as weight gain, sedation, extrapyramidal symptoms, and a high rate of symptom recurrence after discontinuation (8–10). These limitations highlight the urgent need to investigate the mechanisms underlying TD and explore other effective treatment strategies, as emphasized by the American Academy of Neurology (8).

Accumulating evidence suggests that neuroinflammation plays a crucial role in TD (11–13). Previous studies have identified close associations among streptococcal infections, allergic conditions, and tic symptoms (14, 15). Elevated levels of pro-inflammatory cytokines, such as interleukin-1*β* (IL-1*β*), tumor necrosis factor alpha (TNF-*α*), and interleukin-6 (IL-6) have been reported in patients with TD, with TNF-*α* showing the maximum increase, during tic exacerbations (16–18). Neuroimaging studies have provided direct evidence of neuroinflammatory activation in patients with TD, demonstrating microglial activation in the basal ganglia (19). A recent study found a higher prevalence of proinflammatory states in mothers of children with tics, and transcriptomic data implicated that innate immune signaling pathways may correlate maternal inflammation to the childhood tics (20). Although neuroinflammation is well-documented in TD, the specific inflammatory mechanisms involved in its pathogenesis remain unclear.

The gut microbiota modulates the host intestinal immune system, which communicates with the central nervous system via the gut-brain axis (21, 22). Distinct compositional shifts occur in the gut microbiota of TD individuals compared to controls, with a notable accumulation of pro-inflammatory bacteria in TD, although the literature does not present a unified perspective on differences in *α*- or *β*-diversity (23, 24). Gut dysbiosis may facilitate the translocation of proinflammatory factors into the circulation and subsequently into the brain, thereby influencing neuroimmune homeostasis (25). The toll-like receptor 4 (TLR4)/myeloid differentiation primary response 88 (MyD88)/nuclear factor-kappa B (NF-*κ*B) signaling pathway constitutes a crucial molecular nexus within the gut-brain axis. Recent studies on nervous system disorders have demonstrated that the activation of gut TLR4 and its downstream signaling pathways contributes to intestinal inflammation. This inflammation can then translocate into the systemic circulation, reach the central nervous system, and activate central TLR4 signaling to exacerbate neuroinflammation (26). Whether gut microbial dysbiosis contributes to TD pathogenesis via the TLR4/MyD88/NF-*κ*B signaling pathway remains unclear. Systematic elucidation of the gut-brain axis and its underlying mechanisms is of great significance for advancing our understanding of TD pathogenesis and developing novel therapeutic strategies.

Traditional Chinese medicine (TCM), which emphasizes holistic principles and syndrome differentiation-based treatments, has demonstrated unique advantages in the clinical management of TD. Its therapeutic efficacy has not only been endorsed by the China National Administration of Traditional Chinese Medicine but has also been acknowledged in international clinical guidelines, including the 2019 Practice Guideline Recommendations Summary by the American Academy of Neurology and the 2022 European Clinical Guidelines for Tourette Syndrome and Other Tic Disorders (9, 10). Yinhua Gouteng Decoction (YHGTD) is a novel Chinese herbal formulation developed as an optimized combination of the classical prescriptions of Yin-Qiao Powder and Tianma Gouteng Decoction. Traditionally, Yin-Qiao Powder has been used to treat infectious diseases. Modern pharmacological studies have confirmed that it can exert anti-inflammatory effects by inhibiting the NF-κB signaling pathway and reducing the expression of pro-inflammatory cytokines, such as IL-6 and TNF-α (27). Tianma Gouteng Decoction is known for its antispasmodic properties, and pharmacological research has suggested that it can protect dopaminergic neuronal function (28). By integrating these anti-inflammatory and neuroprotective characteristics, YHGTD exhibits the potential for multi-target and synergistic regulation in TD treatment. Clinical studies have demonstrated that YHGTD effectively alleviates tic symptoms in children, including throat clearing, tongue protrusion, and lip puckering (29, 30). It has also been granted a national invention patent (Patent No. 201710319365.4) that provides a solid foundation for further research and clinical application (31). Our previous transcriptomic analysis revealed that YHGTD may exert its therapeutic effects by regulating the dopaminergic system and immune-related pathways (32); however, the specific mechanisms remain to be fully elucidated.

**Figure 1:**
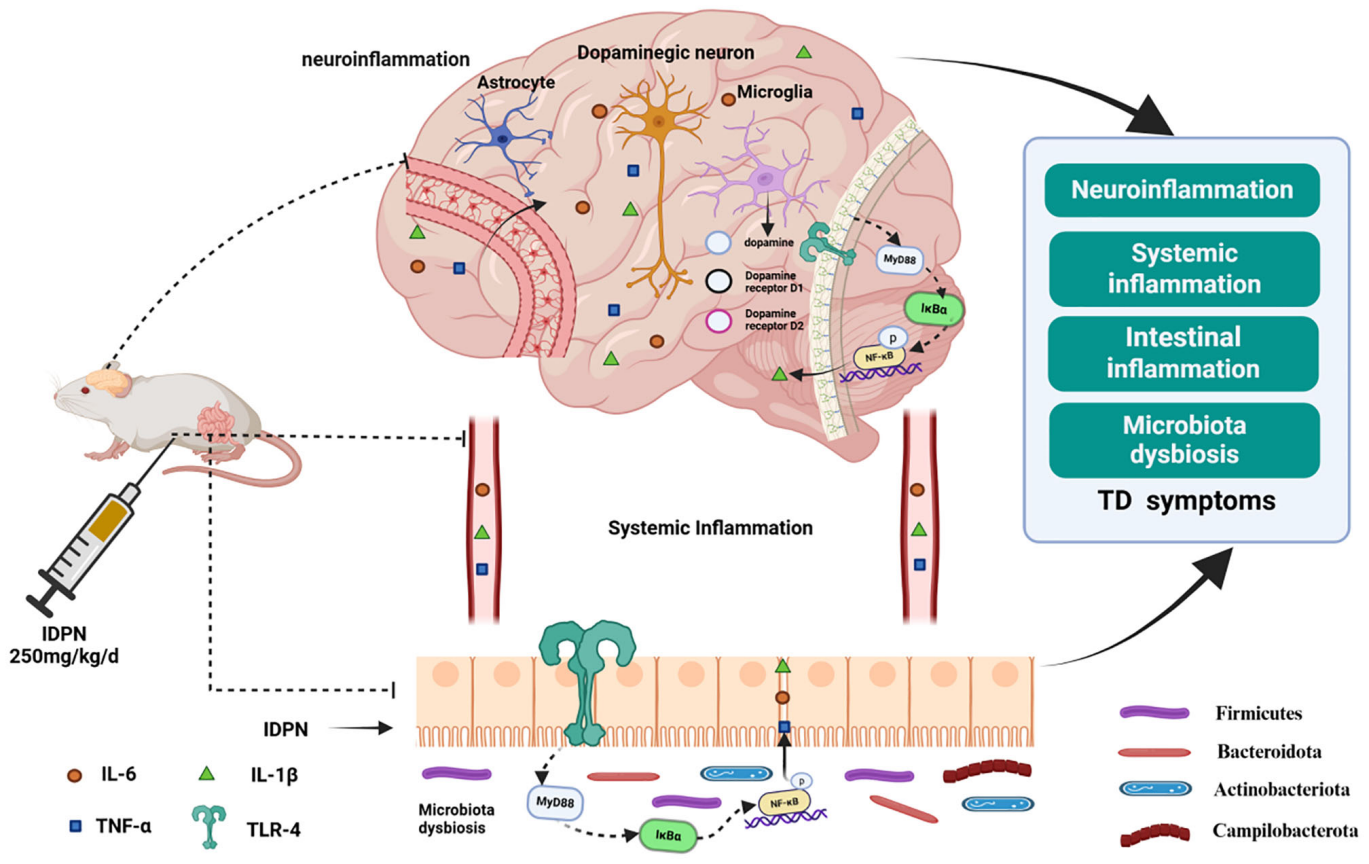
Mechanism schematic of YHGTD modulating neuroinflammation in TD.

In the present study, we employed a 3,3’-iminodipropionitrile (IDPN)-induced TD rat model to systematically investigate the therapeutic effects of YHGTD, with a focus on behavioral assessments, dopaminergic dysfunction, neuroinflammation, and gut-brain axis signaling. We aimed to provide experimental evidence and theoretical support for elucidating the pathophysiology of TD and promoting the development of novel Chinese herbal medicines. A schematic overview of the proposed mechanism is presented in **Figure 1**.

## 2 Materials and methods

### 2.1 Drugs

YHGTD granules (Sichuan New Green Pharmaceutical Technology Development Co., Ltd., Chengdu, China) were obtained from the Shanghai Municipal Hospital of Traditional Chinese Medicine. YHGTD consisted of the following herbal components: *Lonicera japonica Thunb.* 9 g; *Forsythia suspensa (Thunb.) Vahl* 9 g; *Uncaria rhynchophylla (Miq.) Jacks.* 9 g; *Gastrodia elata Blume* 9 g; *Scrophularia ningpoensis Hemsl.* 6 g; *Paeonia lactiflora Pall.* 9 g; *Magnolia biondii Pamp.* 6 g; *Xanthium sibiricum Patr.* 6 g; *Bombyx mori Linnaeus* 6 g; *Buthus martensii Karsch* 3 g; *Lycopodium japonicum Thunb.* 9 g; *Tribulus terrestris L.* 9 g; *Glycyrrhiza uralensis Fisch.* 6 g. Tiapride tablets were purchased from Jiangsu Enhua Pharmaceutical Co., Ltd (0.1 g/tablet, batch number: H32025477, Jiangsu, China). IDPN was purchased from Sigma-Aldrich (Cat# 317306, St. Louis, MO, USA).

### 2.2 Animal model establishment and experimental design

Sixty 4-week-old male SPF Sprague-Dawley (SD) rats were provided by Slake Experimental Animal Co., Ltd (animal license no. SCXK 2022-0004, Shanghai, China). All experimental procedures were approved by the Ethics Committee of the Shanghai Municipal Hospital of Traditional Chinese Medicine (Approval No. 2022032). Rats were housed in the animal facility under controlled conditions (22 ± 2°C, 50–60% humidity, 12-h light/dark cycle), with ad libitum access to sterilized food and water. After a 1-week week acclimatization period, rats were randomly assigned to six groups: control, model, tiapride (23.80 mg/kg), low-dose YHGTD (11.78 g/kg), medium-dose YHGTD (23.56 g/kg), and high-dose YHGTD (47.12 g/kg).

Rats received intraperitoneal injections of IDPN (250 mg/kg/day) for 7 consecutive days to establish the TD model, whereas control rats were administered an equal volume of sterile 0.9% saline. A rat was considered successfully modeled when both its motor and stereotyped behavior scores were ≥ 2, as defined in **Table 1**. Following model establishment, rats were orally administered YHGTD or tiapride once daily by gavage at a volume of 10 mL/kg for 4 weeks. YHGTD granules were dissolved in sterile 0.9% saline to prepare working solutions corresponding to the low-, medium-, and high-dose treatments. Tiapride tablets were crushed into a fine powder and dissolved in sterile 0.9% saline. Fresh drug solutions were prepared daily before administration. The control and model groups received equal volumes of sterile 0.9% saline throughout the treatment period. Body weight was recorded weekly throughout the experiment.

**Table 1:** Motor and stereotypic behavior scoring criteria.

Score	Motor behavior	Stereotypic behavior
0	Quiet or normal activity	No stereotypic behavior
1	Hyperactivity	Rotation behavior (clockwise or counterclockwise)
2	Increased exploratory behavior with discontinuous sniffing	Excessive vertical head and neck movements
3	Continuous running	Excessive vertical head and neck movements accompanied by rotation
4	Continuous running with jumping	Lateral head swinging along with excessive vertical head and neck movements

**Figure 2:**
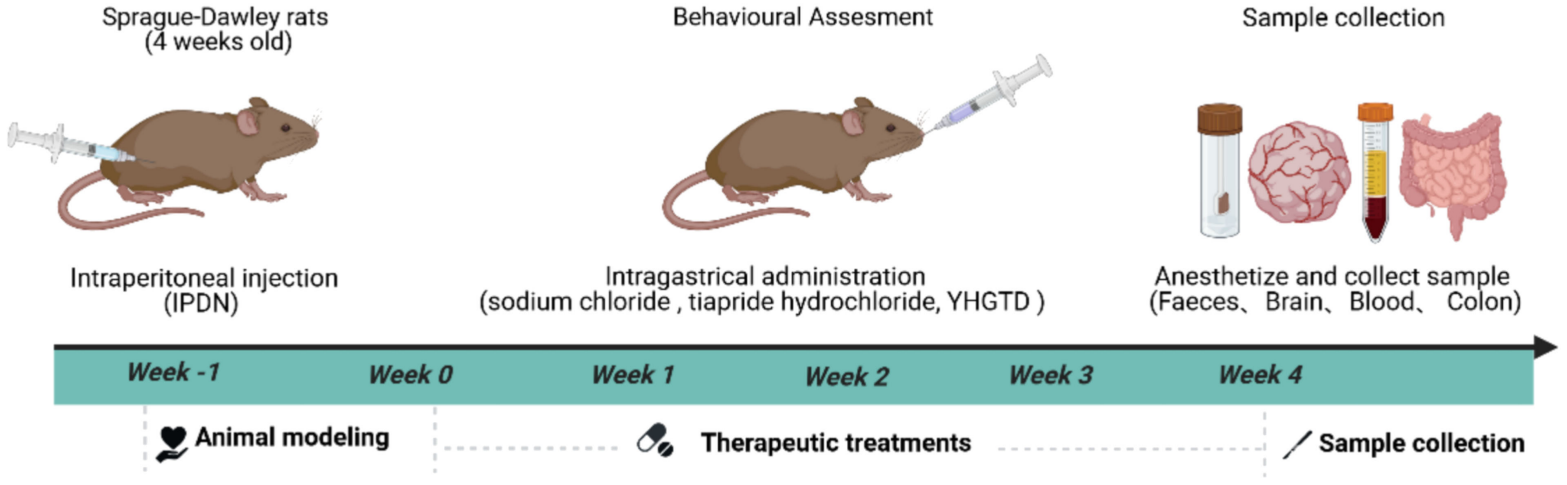
The flow chart of animal treatments.

At the end of the 4-week intervention, fecal samples were collected, and all rats were euthanized under anesthesia. The serum, brain, and colon tissues were harvested for further analysis. An overview of the experimental design is presented in **Figure 2**.

### 2.3 Reagents and chemicals

Antibodies against DRD1 (ab81296), DRD2 (ab85367), p-I*κ*B*α* (ab133462), I*κ*B*α* (ab32518), MyD88 (ab219413), GAPDH (ab181602), *β*-actin (ab8226), and Iba-1 (ab178846) were obtained from Abcam (Cambridge, UK). The NF-*κ*B p65 antibody was purchased from Cell Signaling Technology (CST, Cat# D14E12, Danvers, MA, USA), and the TLR4 antibody from Affinity Biosciences (Cat# AF7017, Cincinnati, OH, USA). ELISA kits for IL-1*β* (RA20020), TNF-*α* (RA20035), and IL-6 (RA20607) were purchased from Bioswamp (Wuhan, China).

### 2.4 Behavioral testing

#### 2.4.1 Motor and stereotypic behavior scoring

Motor and stereotypical behaviors were assessed at baseline (week 0), and after 4 weeks of treatment, 1 h after administration. Each rat was individually placed in an observation cage under dim lighting in a quiet environment. After a 5-min adaptation period, the behaviors were observed for 5 min. All behavioral sessions were video-recorded. Two trained observers independently scored the behaviors in a double-blind manner according to the criteria listed in **Table 1**, and the average score was used for the final analysis.

#### 2.4.2 Spontaneous activity assessment

Spontaneous activity tests were conducted 0, 1, 2, 3, and 4 weeks after administration. The rats were placed in a black activity box (50 × 50 cm), and locomotor activity was tracked using the VisuTrack Animal Behavior Analysis System (Xinruan, China). Parameters, including total travel distance, rest time, and movement trajectories, were recorded. Each test was conducted in a quiet environment, and each rat was observed for 5 min. After each experiment, the feces were cleaned, and the interior of the experimental box was wiped with 75% ethanol to remove odors and minimize interference.

### 2.5 LC-MS/MS analysis

The striatal tissue was homogenized in pure water at a ratio of 1:2 (w/v). A 30 μL aliquot of the homogenate was mixed with 90 μL of acetonitrile containing tetraethylammonium (internal standard, 500 ng/mL) and vortexed for 1 min. After centrifugation, the supernatant was collected for liquid chromatography-tandem mass spectrometry (LC-MS/MS) analysis. Dopamine (DA) standard solutions were prepared by dissolving the reference compound in 50% acetonitrile, followed by serial dilution. For calibration, 30 μL of each DA standard solution was treated with internal standard and acetonitrile in the same way as the samples before LC-MS/MS analysis. Chromatographic separation was carried out on an ACQUITY UPLC system (Waters Corporation, Milford, MA, USA) equipped with a BEH Amide column (100 × 2.1 mm, 1.7 μm). The mobile phases were 0.2% acetic acid in 5 mM ammonium acetate (A) and 0.1% formic acid in acetonitrile (B), with the column temperature maintained at 40°C. Mass spectrometric detection was performed in positive ion mode using an electrospray ionization source, with a source voltage of 5500 V and a nitrogen gas pressure of 60 psi.

### 2.6 Real-time quantitative PCR

Total RNA was extracted from the striatal and colonic tissues using TRIzol reagent (Servicebio, Wuhan, China). cDNA was synthesized using HiScript III RT SuperMix for qPCR (Vazyme, Nanjing, Jiangsu, China), according to the manufacturer’s instructions. Real-time quantitative PCR was performed on a 7500 Real-Time PCR System (Thermo Scientific, Waltham, MA, USA) using ChamQ Universal SYBR qPCR Master Mix (Vazyme, Nanjing, Jiangsu, China). The relative mRNA expression levels of *Il-1β*, *Tnf-α*, and *Il-6* were quantified in both the striatum and colon. *Gapdh* was used as the internal control, and relative gene expression was calculated using the 2 ^−ΔΔCt^ method. The specific primer sequences are listed in **Table 2**.

**Table 2:** Primer information.

Genes	Primer sequence 5’→3’	
*Drd1*	Forward primers	GCATGGCTTGGATTGCTACG
	Reverse primers	AGGAGAAATCCCTCTCCGCT
*Drd2*	Forward primers	AGACACCACTCAAGGATGCTG
	Reverse primers	CAATCTTGGCGTGCCCATTC
*Il-1β*	Forward primers	CAGCTTTCGACAGTGAGGAGA
	Reverse primers	TTGTCGAGATGCTGCTGTGA
*Tnf-α*	Forward primers	ATGGGCTCCCTCTCATCAGTTCC
	Reverse primers	GCTCCTCCGCTTGGTGGTTTG
*Il-6*	Forward primers	ACTTCCAGCCAGTTGCCTTCTTG
	Reverse primers	TGGTCTGTTGTGGGTGGTATCCTC
*Gapdh*	Forward primers	GCAAGTTCAACGGCACAGTCAAG
	Reverse primers	CACGACATACTCAGCACCAGCAT

### 2.7 Western blotting

Proteins from the striatal and colonic tissues were extracted using RIPA lysis buffer (Beyotime, Shanghai, China), and protein concentrations were determined using a BCA assay kit (Yeasen, Shanghai, China). Equal amounts of protein (25 μg per sample) were separated on 7.5% or 10% SDS-PAGE gels and transferred to PVDF membranes. Membranes were blocked with a protein-free rapid blocking solution for 15–20 min and then incubated overnight at 4°C with primary antibodies against DRD1 (1:1000), DRD2 (1:400), TLR4 (1:1000), MyD88 (1:1000), I*κ*B-α (1:1000), p-I*κ*B-*α* (1:1000), NF-*κ*B p65 (1:1000), GAPDH (1:6000), and *β*-actin (1:6000). After washing, the membranes were incubated with the appropriate secondary antibodies at room temperature for 1 h. Immunoreactive bands were visualized using enhanced chemiluminescence (ECL, Thermo Scientific, Waltham, MA, USA) and captured with a ChemiDoc™ XRS+ imaging system (Bio-Rad, Hercules, CA, USA). Band intensities were quantified using the Image Lab software.

### 2.8 H&E staining

Striatal tissue was fixed in 4% paraformaldehyde for 48 h, embedded in paraffin, and sectioned into 4 μm thick sections, which were stained with hematoxylin and eosin (H&E). The striatal morphology was examined under a light microscope.

### 2.9 Immunofluorescent staining

Striatal sections were dewaxed and rehydrated, followed by antigen retrieval using trypsin at 37°C for 20 min. Membranes were permeabilized using 0.1% Triton X-100 at room temperature for 10 min. After blocking with 5% BSA at room temperature for 1 h, sections were incubated overnight at 4°C with Iba1 primary antibody (1:1000), then with a fluorescent secondary antibody at room temperature in the dark for 1 hour. Nuclei were counterstained with a spontaneous fluorescence quenching agent containing DAPI for 10 minutes at room temperature in the dark. Finally, the sections were observed under a fluorescence microscope.

### 2.10 ELISA

Serum levels of the inflammatory cytokines IL-1*β*, TNF-*α*, and IL-6 were quantified using commercial ELISA kits, following the manufacturer’s instructions.

### 2.11 Sequencing and bioinformatic analysis of gut microbiota

Total microbial genomic DNA was extracted from fecal samples using the E.Z.N.A^®^Soil DNA Kit (Omega Bio-tek, Norcross, GA, USA) according to the manufacturer’s instructions. The hypervariable V3-V4 region of the bacterial 16S rRNA gene was amplified using the primer pairs 338F (5’-ACTCCTACGGGAGGCAGCAG-3’) and 806R (5’-GGACTACHVGGGTWTCTAAT-3’). DNA quality and concentration were determined using 1.0% agarose gel electrophoresis and a NanoDrop-2000 spectrophotometer (Thermo Scientific, Waltham, MA, USA). Sequencing was performed on an Illumina NextSeq 2000 platform (Illumina, San Diego, CA). Raw sequence data were quality filtered using fastp (v0.20.0) and merged with FLASH (v1.2.11). Sequence clustering, chimera removal, and operational taxonomic units (OTUs) picking at 97% similarity were performed using UPARSE (v7.1). Taxonomic classification was conducted using the RDP Classifier (v2.2) against the Silva 16S rRNA gene database (v138) at a 70% confidence threshold. All data analyses were performed on the Majorbio Cloud Platform (https://cloud.majorbio.com). Group differences in *α*-diversity indices were evaluated using the Kruskal-Wallis test. Community structure variation was examined through principal coordinate analysis and non-metric multidimensional scaling based on Bray-Curtis distance metrics, with statistical significance assessed using the Adonis test. Differential abundance analyses at the phylum and family levels were performed using the Kruskal–Wallis test combined with false discovery rate correction. The sequencing data were deposited in the NCBI Sequence Read Archive (SRA) under BioProject accession number PRJNA1245788.

### 2.12 Statistical analysis

Data are expressed as mean ± standard deviation (SD). Travel distance data from spontaneous activity experiments were analyzed using a two-factor repeated-measures analysis of variance (ANOVA). Other group comparisons were performed using one-way ANOVA or nonparametric testing depending on data distribution and variance homogeneity. Correlations between behavioral and molecular parameters were assessed using Spearman’s rank correlation. *P*<0.05 was considered statistically significant. Statistical analyses were conducted using IBM SPSS Statistics (version 26.0) and GraphPad Prism (version 10.0).

## 3 Results

### 3.1 YHGTD ameliorated tic-like behaviors in IDPN-induced TD rats

**Figure 3:**
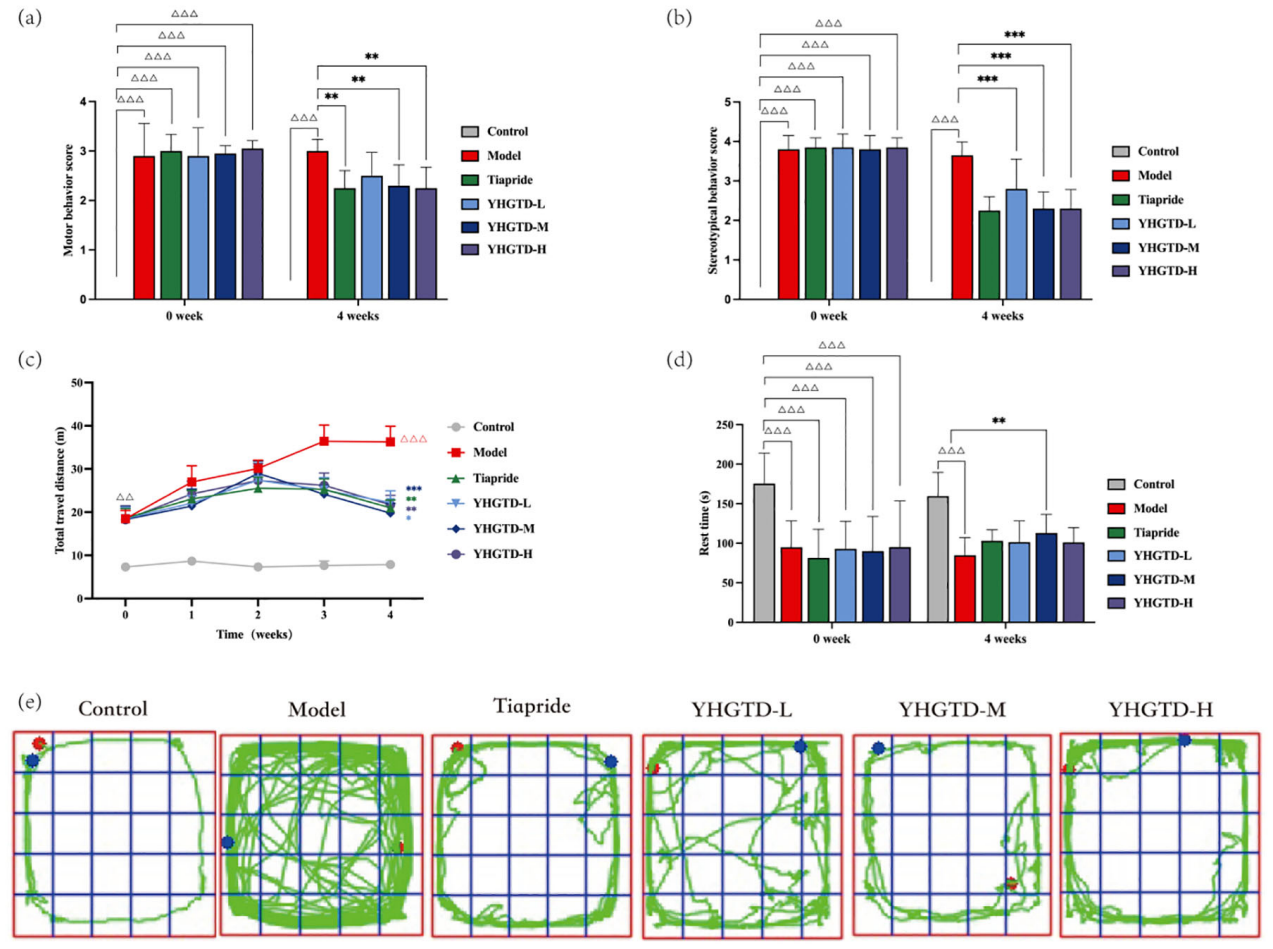
YHGTD ameliorates tic-like behaviors in IDPN-induced TD rats. **(a)** Motor behavior score; **(b)** Stereotypical behavior score; **(c)** Total travel distance; **(d)** Rest time; **(e)** Typical movement trajectories. n = 10 per group. Compared with the control group, ^△△^*P*<0.01, ^△△△^*P*<0.001; Compared with the model group, ^*^P<0.05, ^**^P<0.01, ^***^P<0.001.

Motor and stereotypical behavior scores were evaluated to assess the therapeutic efficacy of YHGTD. At week 0, both scores in the model group were ≥ 2, significantly higher than those in the control group (*P*<0.001, **Figures 3a, b**). After 4 weeks of treatment, rats administered tiapride, medium-dose YHGTD, or high-dose YHGTD showed significant reductions in both motor behavior score (*P*<0.01, **Figure 3a**) and stereotypic behavior score (*P*<0.001, **Figure 3b**). Although a downward trend was observed in the low-dose YHGTD group, the differences compared with the model group were not statistically significant (*P* > 0.05).

Spontaneous locomotor activity was also assessed. At week 0, the model and treatment groups exhibited significantly increased total movement distances (*P <*0.01) and decreased rest times (*P <*0.001) compared to the controls. Notably, while the movement distances in all treatment groups peaked at week 2 and then declined, those in the model group peaked at week 3 before plateauing. After 4 weeks of treatment, all intervention groups exhibited significant reductions in total movement distance compared to the model group, with the medium-dose YHGTD group demonstrating the greatest effect (*P*<0.001) and a significant increase in rest time (*P*<0.01). Other treatment groups also showed reduced movement distances (*P <*0.05), but their rest times were not significantly different from those of the model group (*P* > 0.05) (**Figures 3c, d**). Representative movement trajectory maps indicated that rats in the control, tiapride, and medium-dose YHGTD groups primarily exhibited peripheral movement patterns and rarely entered the central area. Rats in the low-dose and high-dose YHGTD groups also showed predominantly peripheral trajectories but entered the central area more frequently than those in the control or medium-dose groups. However, the model group showed a marked increase in the central movement and more disorganized and scattered trajectories (**Figure 3e**).

### 3.2 YHGTD attenuated striatal pathological damage in IDPN-induced TD rats

**Figure 4:**
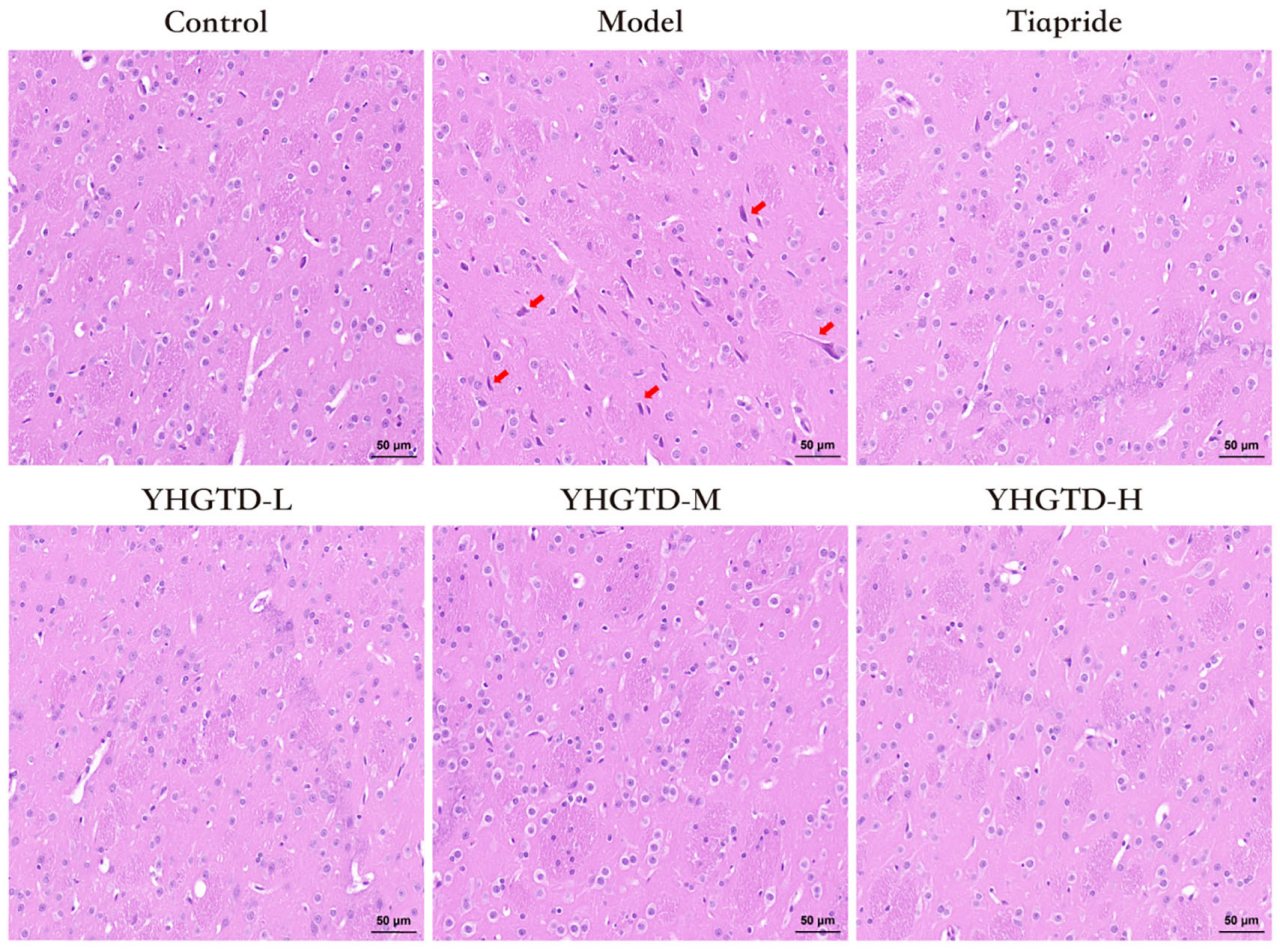
YHGTD attenuates striatal pathological damage in IDPN-induced TD rats. n = 3 per group. Red arrow indicates typical neuronal injury. Scale bar = 50 μm.

H&E staining was performed to examine striatal neuronal architecture. The striatal region of the control group displayed normal histological features, including abundant neurons with round, well-defined cell bodies, clear nuclear boundaries, and prominent nucleoli, with no evidence of neuronal degeneration, glial proliferation, or inflammatory cell infiltration. In contrast, the model group exhibited marked neuronal injury, including degenerative and necrotic changes characterized by nuclear pyknosis, cytoplasmic eosinophilia, nuclear dissolution or vacuolation, and a blurred cellular architecture. Notably, all treatment groups demonstrated substantial histopathological improvements compared with the model group. Neurons in these groups showed more clearly defined structures with only mild pyknosis and slightly intensified nuclear staining (**Figure 4**).

### 3.3 YHGTD regulated DA and dopamine receptors in the striatum of IDPN-induced TD rats

**Figure 5:**
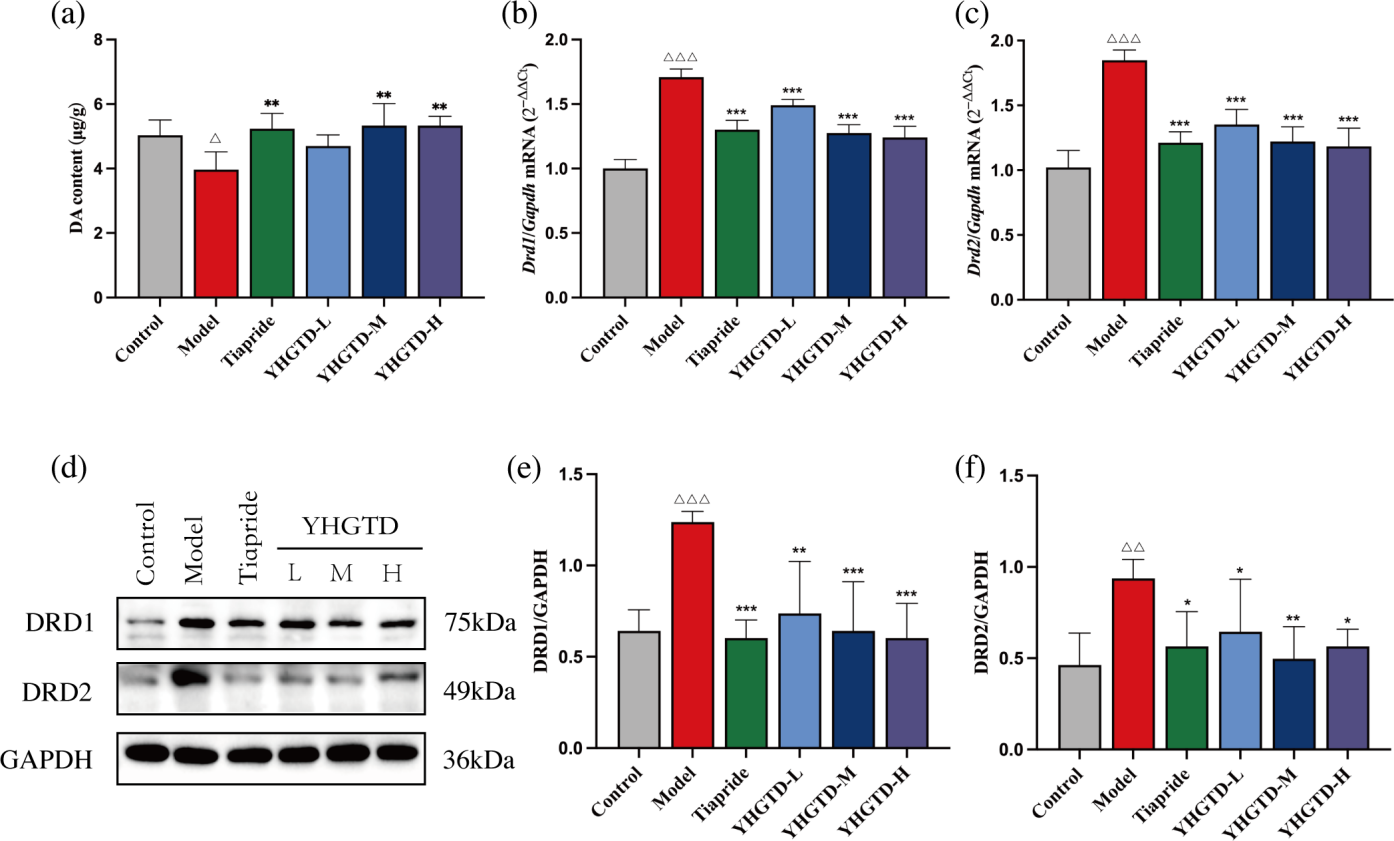
YHGTD regulates DA and DRs in the striatum of IDPN-induced TD rats. **(a)** DA content; **(b)** Relative expression of *Drd1* mRNA; **(c)** Relative expression of *Drd2* mRNA; **(d)** Representative Western blot images of DRD1 and DRD2 proteins; **(e)** Quantification of DRD1 protein expression; **(f)** Quantification of DRD2 protein expression. n = 3–6 per group. Compared with the control group, ^△△^*P*<0.01, ^△△△^*P*<0.001; Compared with the model group, ^*^P<0.05, ^**^P<0.01, ^***^P<0.001.

Dysregulation of dopaminergic signaling in the striatum represents a significant neurobiochemical pathological basis for TD. To evaluate the effect of YHGTD on striatal dopaminergic system function in TD rats, we first measured the DA content in the striatum using LC-MS/MS. The DA levels in the model group were significantly lower than those in the control group (*P*<0.05, **Figure 5a**). In contrast, DA levels were significantly elevated in the tiapride, medium-dose YHGTD, and high-dose YHGTD groups compared to those in the model group (*P*<0.05, **Figure 5a**). Although the low-dose YHGTD group exhibited an upward trend, the difference was not statistically significant (*P* > 0.05).

Next, we assessed the relative mRNA expression of *Drd1* and *Drd2* in the striatum using qPCR. The model group showed significantly increased *Drd1* and *Drd2* mRNA expression levels compared to the control group (*P*<0.001, **Figures 5b, c**). Treatment with tiapride or YHGTD (at all doses) significantly reduced *Drd1* and *Drd2* mRNA expression compared to the model group (*P*<0.001, **Figures 5b, c**).

Western blotting was performed to detect the expression of DRD1 and DRD2 proteins in the striatum. Consistent with the qPCR results, the model group exhibited significantly elevated DRD1 and DRD2 protein levels compared to the control group (*P <*0.01). Both tiapride and YHGTD treatments significantly downregulated DRD1 and DRD2 protein expression compared to the model group (*P <*0.05, **Figures 5d-f**).

### 3.4 YHGTD inhibited microglial activation and pro-inflammatory cytokine release in the striatum of IDPN-induced TD rats

**Figure 6:**
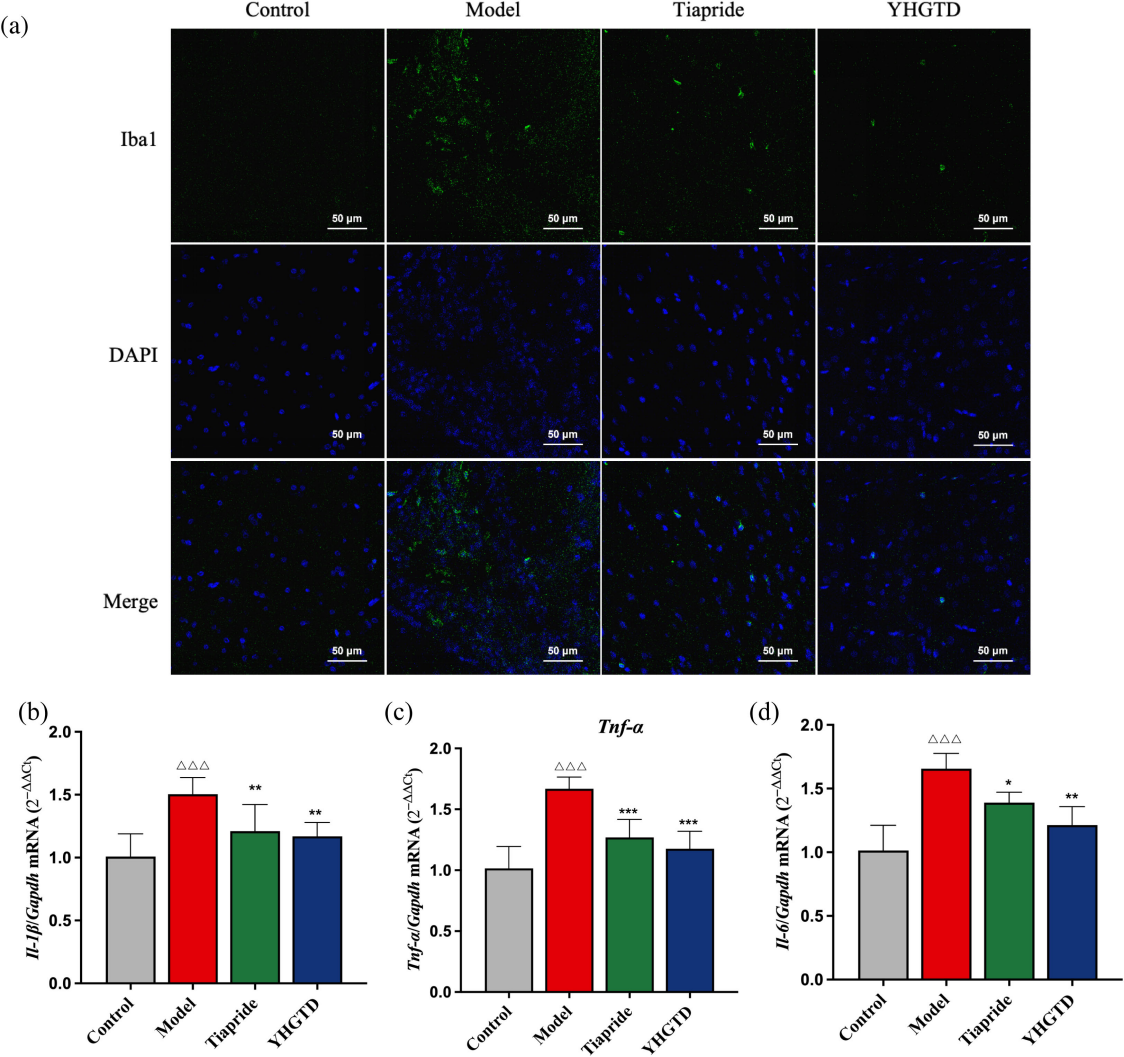
YHGTD inhibits microglial activation and pro-inflammatory cytokine release in the striatum of IDPN-induced TD rats. **(a)** Iba1 immunofluorescence staining; **(b)** Relative expression of *Il-1β* mRNA; **(c)** Relative expression of *Tnf-α* mRNA; **(d)** Relative expression of *Il-6* mRNA; **(e)** Correlation heatmap of striatal biomarkers and behavioral parameters. n = 3–6 per group. Compared with the control group, ^△△△^*P*<0.001; Compared with the model group, *P<0.05, **P<0.01, ***P<0.001.

Based on the experimental results, a medium dose of YHGTD was identified as the most effective and was thus selected for subsequent mechanistic studies. To investigate the anti-inflammatory effects of YHGTD in the TD model, we first assessed microglial activation in the striatum using immunofluorescence staining for Iba1, a specific microglial marker. Compared with the control group, the model group exhibited a significant increase in Iba1 expression, indicating microglial activation. Treatment with YHGTD or tiapride significantly reduced Iba1 expression (**Figure 6a**).

We then measured the relative mRNA expression levels of *Il-1β*, *Tnf-α*, and *Il-6* in the striatum. The model group showed significantly elevated expression of all three pro-inflammatory cytokines compared with the control group (*P <*0.001, **Figures 6b**–**d**). Following treatment with YHGTD or tiapride, the expression levels of *Il-1β*, *Tnf-α*, and *Il-6* were significantly reduced (*P <*0.05, **Figures 6b**–**d**).

Correlation analysis revealed that motor behavior score, stereotypic behavior score, and total travel distance were positively associated with striatal levels of *Drd1*, *Drd2*, *Il-1β*, *Tnf-α*, and *Il-6 (r*>0.6, *P <*0.05). Conversely, total travel distance showed a significant negative correlation with striatal DA levels (*r<*-0.6, *P <*0.05), and rest time was negatively correlated with *Drd1*, *Drd2*, *Il-1β*, *Tnf-α*, and *Il-6* in the striatum (*r<*-0.6, *P <*0.05).

### 3.5 YHGTD reduced pro-inflammatory cytokine release in the serum and colon of IDPN-induced TD rats

**Figure 7:**
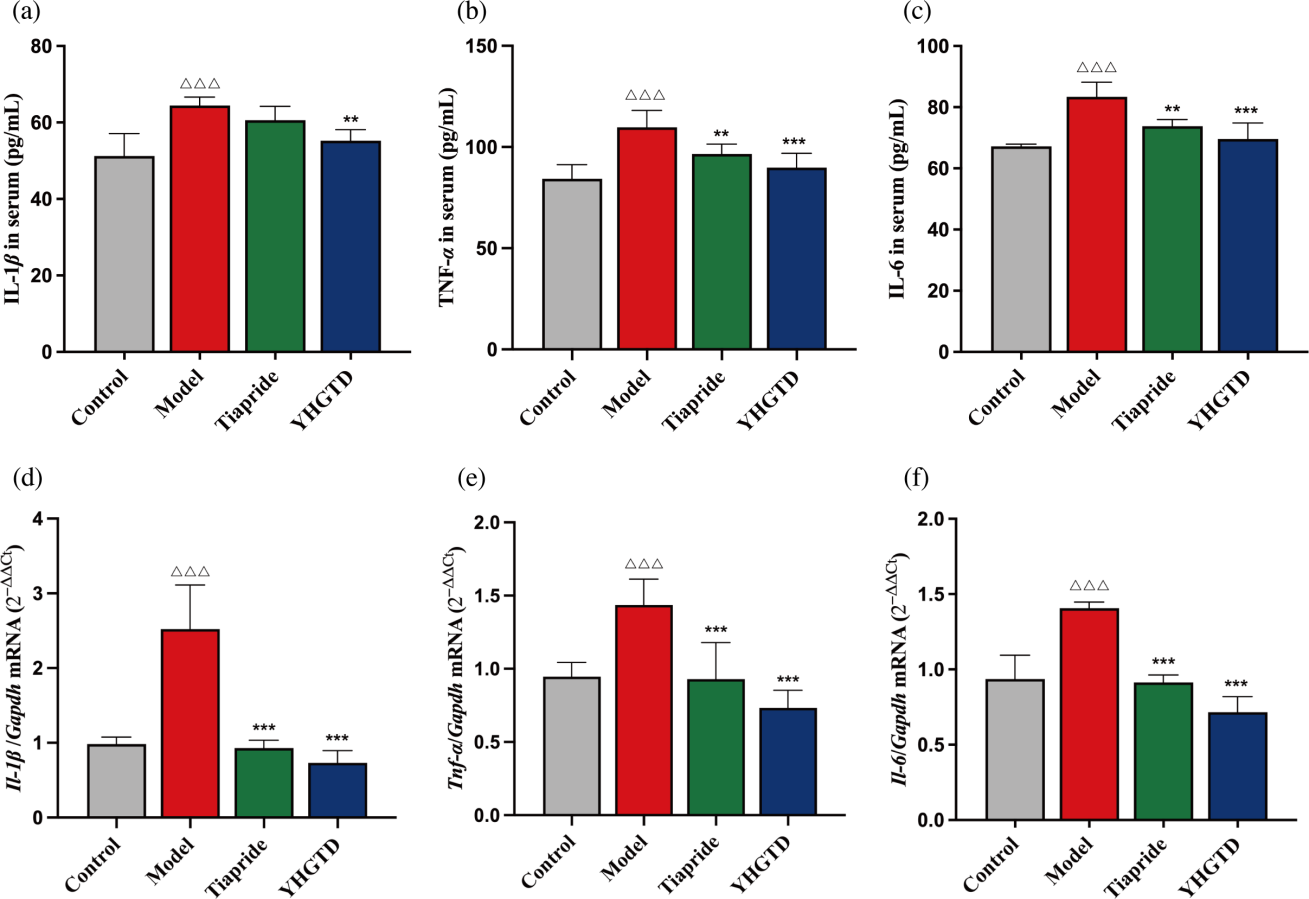
YHGTD reduces pro-inflammatory cytokine release in the serum and colon of IDPN-induced TD rats. **(a)** Serum IL-1*β* levels; **(b)** Serum TNF-*α* levels; **(c)** Serum IL-6 levels; **(d)** Relative expression of *Il*-*1β* mRNAin the colon; **(e)** Relative expression of *Tnf*-*α* mRNA in the colon; **(f)** Relative expression of *Il*-*6* mRNA in the colon. n = 3 per group. Compared with the control group, ^△△△^*P*<0.001; Compared with the model group, ^**^P<0.01, ^***^P<0.001.

To evaluate peripheral inflammation in TD rats, we first measured serum levels of IL-1β, TNF-α, and IL-6. Serum concentrations of all three cytokines were significantly higher in the model group than in the control group (*P <*0.001). Treatment with tiapride or YHGTD significantly reduced serum cytokine levels (*P <*0.01, **Figures 7a**–**c**). In parallel, we assessed the relative mRNA expression of *Il-1β*, *Tnf-α*, and *Il-6* mRNA in the colon. The model group showed markedly increased colonic expression of these cytokines (*P <*0.001), which was significantly decreased following treatment with tiapride or YHGTD (*P <*0.001, **Figures 7d**–**f**).

### 3.6 YHGTD modulated gut microbial dysbiosis in the IDPN-induced TD rats

Given the observed intestinal inflammation in TD rats, we further examined the effects of YHGTD on the gut microbiota composition using 16S rRNA sequencing. A total of 2675 OTUs were identified, with 340 unique OTUs in the control group, 198 in the model group, and 519 in the YHGTD group; 1152 OTUs were shared across all groups.

**Figure 8:**
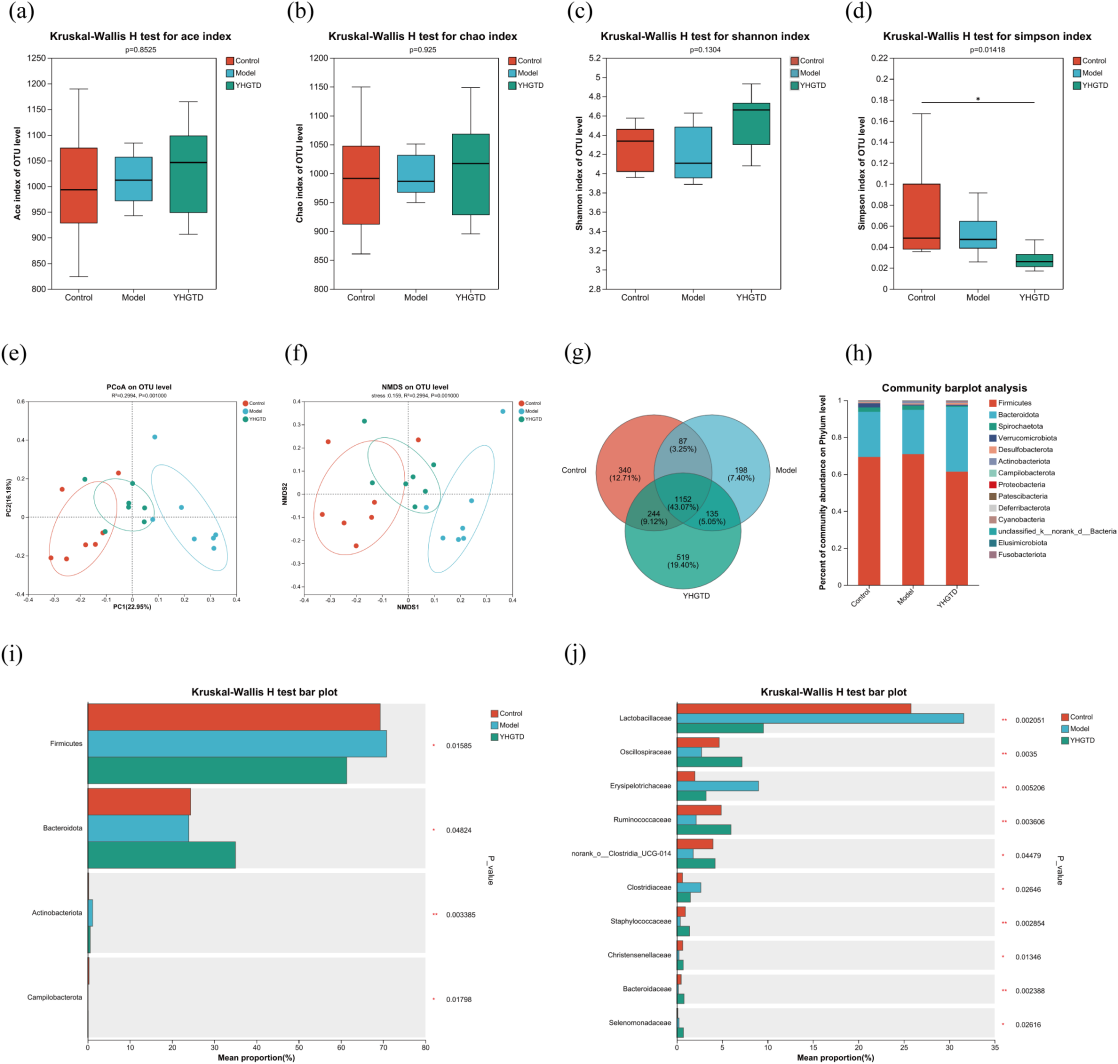
YHGTD modulates gut microbial dysbiosis in the IDPN-induced TD rats. **(a)** Ace index; **(b)** Chao index; **(c)** Shannon index; **(d)** Simpson index; **(e)** PCoA; **(f)** NMDS; **(g)** Venn diagram of OTUs; **(h)** Bar chart of relative abundance at the phylum level; **(i)** Analysis of species differences among the three groups at the phylum level; **(j)** Analysis of species differences among the three groups at the family level. n = 7 per group. Compared with the control group, ^△△^*P*<0.01, ^△△△^*P*<0.001; Compared with the model group, ^*^P<0.05, ^**^P<0.01, ^***^P<0.001.

α-diversity analysis revealed no significant differences in the Ace, Chao, and Shannon indices among the groups, but the Simpson index was significantly higher in the YHGTD group than in controls (*P*<0.05, **Figures 8a**–**d**). PCoA and NMDS analyses demonstrated significant differences in gut microbial community structure between the groups. At the OTU level, the model group showed marked divergence from the controls, whereas the YHGTD group clustered more closely with the controls (*P*<0.01, **Figures 8e**–**f**).

At the phylum level, *Firmicutes* and *Bacteroidota* predominated across all the groups, accounting for over 90% of the total microbiota (**Figure 8h**). Notable differences in the relative abundances of *Firmicutes*, *Bacteroidota*, *Actinobacteriota*, and *Campilobacterota* were observed among the groups (**Figure 8i**). At the family level, differentially abundant taxa included *Lactobacillaceae*, *Oscillospiraceae*, *Erysipelotrichaceae*, *Ruminococcaceae*, and *Clostridiaceae* (**Figure 8j**).

### 3.7 YHGTD suppressed TLR4/MyD88/NF-κB pathway activation in the striatum and colon of IDPN-induced TD rats

Both neuroinflammation and peripheral inflammation were evident in the TD rats, as reflected by the increased levels of intestinal inflammatory cytokines and pro-inflammatory bacteria. Given that the TLR4 signaling pathway is closely associated with neuroinflammation, intestinal inflammation, and microbial imbalance, we examined the expression of TLR4 pathway-related proteins in the striatum and colon.

**Figure 9:**
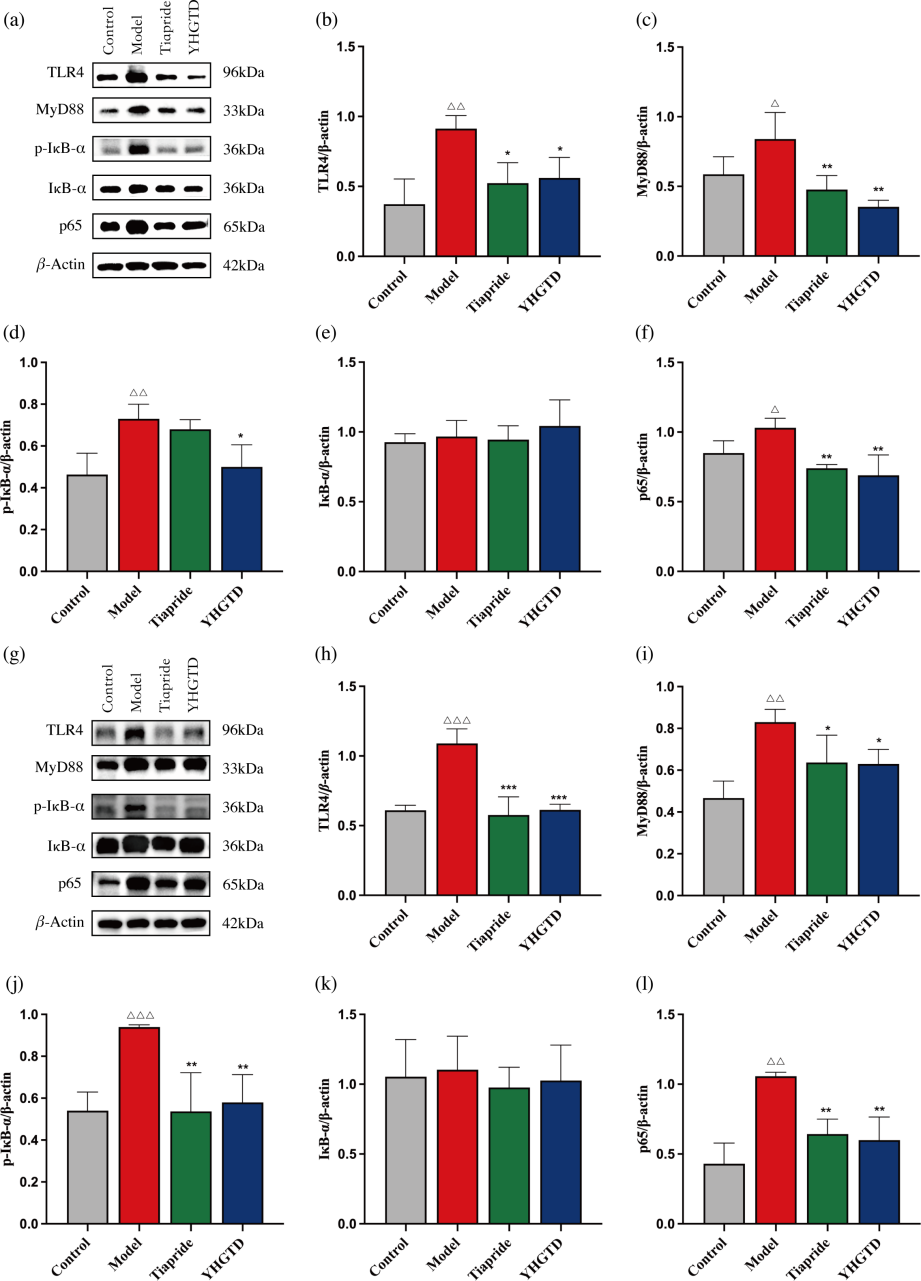
YHGTD suppresses TLR4/MyD88/NF-*κ*B pathway activation in the striatum and colon of IDPN-induced TD rats. **(a)** Representative western blot images of TLR4, MyD88, p-I*κ*B-*α*, I*κ*B-*α* and NF-*κ*B p65 protein in the striatum; **(b-f)** Quantification of TLR4 **(b)**, MyD88 **(c)**, p-I*κ*B-*α***(d)**, I*κ*B-*α***(e)**, NF-*κ*B p65 **(f)** protein expression in the striatum; **(g)** Representative Western blot images of TLR4, MyD88, p-I*κ*B-*α*, I*κ*B-*α* and NF-*κ*B p65 protein in the colon; **(h-l)** Quantification of TLR4 **(h)**, MyD88 **(i)**, p-I*κ*B-*α***(j)**, I*κ*B-*α***(k)**, NF-*κ*B p65 **(l)** protein expression in the colon. n = 3 per group. Compared with the control group, ^△△^*P*<0.01, ^△△△^*P*<0.001; Compared with the model group, ^*^P<0.05, ^**^P<0.01, ^***^P<0.001.

In the striatum, the model group showed significantly elevated expression of TLR4, MyD88, p-I*κ*B*α*, and NF-*κ*B p65 proteins compared to the control group (*P*<0.05). YHGTD treatment markedly reduced the expression of these proteins (*P*<0.05), while tiapride also significantly decreased TLR4, MyD88, and NF-*κ*B p65 expression (*P*<0.05). The reduction in p-I*κ*B*α* in the tiapride group did not reach statistical significance (*P* > 0.05), and there was no significant difference in I*κ*B*α* expression among groups (*P* > 0.05) (**Figures 9a**–**f**).

Similarly, in colon samples, TLR4, MyD88, p-IκBα, and NF-κB p65 protein levels were significantly increased in the model group (*P*<0.01) and were significantly reduced after treatment with YHGTD or tiapride (*P*<0.05). Again, IκBα expression did not differ significantly among groups (*P* > 0.05) (**Figures 9g**–**l**).

## 4 Discussion

TDs are characterized by tics, manifesting as a wide range of repetitive and involuntary movements or vocalizations. The severity, type, and complexity of symptoms vary significantly among individuals. Although the pathophysiology of TD is not fully understood, it is widely accepted that genetic, environmental, and neurophysiological factors contribute to its development. At the neural network level, tics are primarily attributed to inhibitory dysfunction within the sensorimotor cortico-basal ganglia circuit, particularly involving abnormalities in the striatal inhibitory microcircuitry. Dysregulation of DA transmission, including aberrant DA release and increased sensitivity to or upregulation of DRs, plays a central role (6, 7, 33). Based on the above mechanism, in the present study, the widely used TD-inducing neurotoxin IDPN, which reduces striatal DA levels and enhances DR sensitivity, was used to elicit classic tic-like behaviors in rodents (34, 35). One week after IDPN administration, stereotypical behaviors were successfully induced in SD rats, including head nodding, retrograde movement, and abnormal circling, along with increased locomotor distance, decreased rest time, and disrupted movement trajectories. After 4 weeks of YHGTD treatment, IDPN-induced TD rats exhibited significant reductions in both motor and stereotypical behaviors, with a marked decrease in total movement distance and movement trajectories that approached those of the control group. Moreover, YHGTD markedly ameliorated morphological and structural abnormalities in neurons in the striatal region, elevated striatal DA levels, and downregulated DRD1 and DRD2 expression. These findings indicate that YHGTD not only effectively alleviates tic-like symptoms in TD model rats, but also exerts its anti-tic effects by attenuating DR hypersensitivity and stabilizing striatal dopaminergic signaling. Meanwhile, we found that the medium dose, which represents the clinically equivalent dose, exhibited superior therapeutic efficacy and greater behavioral stability, a finding that is consistent with our clinical studies and previous preclinical research (29, 32). Therefore, the medium dose was selected for subsequent mechanistic investigations.

Although the dopamine hypothesis has greatly advanced our understanding of the pathogenesis of TDs, it does not fully account for the diverse pathological features observed in patients. Recent studies have suggested that damage to striatal dopaminergic neurons is closely linked to microglial activation (36, 37). As the resident immune cells of the central nervous system, microglia play a central role in neuroimmune interactions and are pivotal in regulating neuronal excitability and neurotransmitter homeostasis (38, 39). Microglia recognize pathological signals through pattern recognition receptors, such as TLR4, which trigger the cascade release of pro-inflammatory cytokines and exacerbate neural dysfunction (38, 40). Transcriptomic and neuroimaging studies have revealed aberrant microglial activation in the striatal regions of patients with TD (19, 41, 42). Ke et al. (43) used IDPN to induce significant neuroinflammation in the brain. In line with these findings, our study demonstrated pronounced microglial activation in the striatum of the IDPN-induced TD rat model, accompanied by elevated expression levels of IL-1*β*, TNF-*α*, and IL-6. Notably, the inflammatory alterations observed are not restricted to the central nervous system but are also mirrored in peripheral tissues, including the blood and colon. YHGTD administration effectively attenuated these central and peripheral inflammatory responses, as evidenced by reduced microglial activation and lower pro-inflammatory cytokine expression. Together, these observations suggest that the anti-tic effects of YHGTD are at least partially mediated through its immunomodulatory actions and the restoration of neural homeostasis, an interpretation further reinforced by correlation analyses linking behavioral improvements with shifts in striatal dopaminergic and inflammatory markers.

Growing evidence indicates that gut microbiota alterations may influence neuroinflammation and neurotransmitter regulation through immune, metabolic, and neural pathways (44–46). Microbiota-gut-brain interactions have also been documented across several neurological disorder studies, supporting a potential link between microbial dysbiosis and neuroinflammatory processes (47–49). In children with TD, Xi et al. (24) further reported enrichment of the pro-inflammatory bacterium Ruminococcus *lactaris*, suggesting that microbial signatures may be linked to host immune and neurotransmitter regulation. Consistent with previous reported IDPN-induced models, we also observed significant alterations in gut microbial community structure (50, 51). Although *α*-diversity did not differ significantly between the control and model groups, which aligns with the clinical findings of Wang et al. (52), *β*-diversity analysis revealed clear separation between the two groups, while the YHGTD-treated group displayed a microbial community structure similar to that of the controls. Several phyla differed among groups, included *Firmicutes*, *Bacteroidota*, *Actinobacteriota*, and *Campilobacterota*, which aligns with prior observations in TD populations (52). Some studies have linked the *Firmicutes*/*Bacteroidota* ratio to the onset age of TD and inflammatory bowel disease (23). A significant increase in *Actinobacteriota* abundance has been observed in patients with ankylosing spondylitis, accompanied by elevated levels of inflammatory cytokines, such as IL-23, IL-17, and IFN-γ (53). At the family level, enrichment of *Clostridiacea* in the TD model group is notable, as this taxon has been associated with TLR4 expression and pro-inflammatory responses in ulcerative colitis (54). Together, these findings support a potential association between microbial dysbiosis and neuroinflammation in TD. The observed improvements in tic-like behaviors following YHGTD treatment may therefore be related to its modulatory effects on gut microbial composition, particularly the reduction of pro-inflammatory taxa.

TLR4 is a key receptor in the innate immune response and is widely expressed in the brain and gut (55). Upon recognition of specific ligands, the extracellular domain of TLR4 interacts with the Toll/IL-1 receptor domain of the adaptor protein MyD88, triggering early NF-*κ*B activation and the subsequent release of pro-inflammatory mediators (56, 57). Substantial studies indicates the TLR4 pathway plays a crucial role in microbiota-gut-brain communication, and TLR4 knockout has been shown to alleviate gut inflammation and neuroinflammation (58–60). Furthermore, recent studies have reported that several TCM formulas downregulate the TLR4 signaling pathway in the colon and brain, inhibit neuroinflammation and improve depressive-like symptoms or cognitive impairments (61, 62). These finding suggest that modulation of TLR4 signaling may be an important mechanism underlying the neuroprotective effects of TCM through the microbiota-gut-brain axis. In the present study, activation of the TLR4/MyD88/NF-*κ*B signaling pathway was significantly increased in both the striatum and colon of IDPN-induced TD rats, whereas YHGTD intervention reversed these changes. These results indicate that YHGTD may exert its anti-tic effects, at least in part, by modulating peripheral and central inflammation through TLR4-mediated downstream signaling pathways.

This study confirmed the therapeutic effects of YHGTD on tic behavior and its regulatory influence on the dopaminergic system in IDPN-induced TD rats. Additionally, we provide preliminary insights into how YHGTD may alleviate neuroinflammation, as reflected by reduced microglial activation and decreased expression of pro-inflammatory cytokines, including IL-1*β*, TNF-*α*, and IL-6. Furthermore, YHGTD alleviated gut microbiota dysbiosis in the TD model, accompanied by reduced pro-inflammatory cytokines levels and attenuated activation of the TLR4/MyD88/NF-*κ*B signaling pathway in both the gut and brain. These findings suggest that gut microbiota may contribute to the therapeutic actions of YHGTD and highlight a promising direction for future mechanistic and therapeutic investigations.

However, several limitations should be acknowledged. First, the IDPN model and the exclusive use of male rats do not fully capture the complexity of human TD. Future studies should validate these results across multiple TD models to enhance the robustness of the conclusions. Second, this study only indicates a potential association involving the gut microbiota, more systematic functional investigations, such as fecal microbiota transplantation or analysis of bacterial metabolites, are needed to clarify the mechanistic contribution of the gut microbiota. Third, the absence of gene-knockout models or pharmacological inhibition of the TLR4/MyD88/NF-*κ*B axis prevents us from establishing the causal role of this pathway in YHGTD’s anti-tic effects. Moreover, given the multi-component and multi-target nature of TCM formulas, additional signaling pathways are likely to be involved and deserve further exploration to fully characterize their biological activities.

## Data Availability

The data presented in the study are deposited in the NCBI SRA repository, accession number PRJNA1245788.
